# Lactylation modification in cancer: mechanisms, functions, and therapeutic strategies

**DOI:** 10.1186/s40164-025-00622-x

**Published:** 2025-03-08

**Authors:** Mengqi Lv, Yefei Huang, Yansu Chen, Kun Ding

**Affiliations:** 1 School of Public Health, Xuzhou Medical University, Xuzhou, 221004 Jiangsu China; 2 Center for Medical Statistics and Data Analysis, Xuzhou Medical University, Xuzhou, 221004 Jiangsu China; 3 Key Laboratory of Human Genetics and Environmental Medicine, Xuzhou Medical University, Xuzhou, 221004 Jiangsu China

**Keywords:** Lactylation, Cancer, Metabolic reprogramming, Metastasis, Therapy

## Abstract

Cancer remains the leading cause of mortality worldwide, and the emergence of drug resistance has made the identification of new therapeutic targets imperative. Lactate, traditionally viewed as a byproduct of glycolysis with limited ATP-producing capacity, has recently gained recognition as a critical signaling molecule. It plays a key role not only in cancer cell metabolism but also in shaping the tumor microenvironment (TME). Histone lysine lactylation, a newly identified post-translational modification, has been shown to influence a range of cellular processes in cancer. Current research focuses on the mechanisms and functions of histone lactylation in cancer, including its role in gene expression regulation, signal transduction, and protein synthesis. However, despite these advancements, there are still plenty of barriers in the quest to unravel the mechanisms of lactylation modification. The emergence of single-cell and spatial transcriptomics may offer valuable insights for selecting targets. This review provides a comprehensive summary of the mechanisms and the applications of lactylation modification in clinical settings. Through a detailed analysis, we identify the key challenges and limitations that exist in the current research landscape. These insights lay the groundwork for future studies by highlighting promising research directions.

## Background

The rapid advancement of proteomics and genomics technologies has significantly expanded our understanding of the epigenetic landscape in cancer. The wide application of Whole Genome Sequencing (WGS) in cancer research has not only revealed the somatic mutation landscape of epigenetic regulators, but also greatly accelerated the burgeoning research on the link between epigenetics and cancer [1]. Epigenetics refers to the regulation of gene expression through a series of heritable modification mechanisms without altering the DNA sequence of genes, and its main regulatory mechanisms include DNA methylation, histone modifications, non-coding RNA regulations, chromatin remodeling, and nucleosome positioning [2].

Histone modifications are a key component of epigenetic regulation and play a critical role in cancer. On the one hand, tumor metabolites can act as cofactors, donors, or antagonists of epigenetic modification enzymes, thereby influencing histone modifications. On the other hand, epigenetic modifications can regulate cellular metabolism by directly altering the expression of metabolic enzymes and transporter proteins, or influencing the expression of signal transduction and transcription factors [3]. Numerous studies have demonstrated that histone modifications are involved in various tumor cell processes, including proliferation [4], invasion [5], metastasis [6], and metabolic reprogramming [7], etc.

Metabolic alterations are hallmarks of cancer, including increased aerobic glycolysis, elevated glucose uptake, dysregulated glutamine metabolism, and reliance on alternative energy sources [8]. These metabolic changes support the high energy and biosynthetic demands of tumor growth and enable tumor cells to adapt to hypoxic environments [9]. Under normal physiological conditions, lactate plays several important roles, including energy production, maintaining blood glucose levels, and supporting brain metabolism during hypoglycemia [10]. Generally, when the demand for ATP and energy exceeds the supply, the production of lactate increases. The Warburg effect [11] highlights that even under aerobic conditions, glycolysis leads to lactate production, a phenomenon first observed in cultured tumor cells. Although the mechanism behind this was unclear at the time, it is now recognized that lactate plays a crucial role in cancer biology.

Lactate accumulation in tumors can reach concentrations of up to 40 mM, compared to normal tissues. High lactate levels are correlated with increased metastasis during early cancer stages [12]. Besides, accumulation of lactate in the TME acidifies the extracellular environment due to H^+^, which generates a transmembrane electrochemical H^+^ gradient (also known as proton motive force) across the plasma membrane of cancer cells. This gradient facilitates the active uptake of nutrients, thereby promoting tumor progression [13].

One particularly striking study in 2019 revealed a novel function for lactate at the epigenetic level—lactylation of lysine residues. This discovery not only expands the biological roles of lactate but also provides direct evidence of its involvement in tumor signaling [14]. Zhang et al. identified 28 lactylation sites on core histones in human and mouse cells. In addition, the increased histone lactylation observed in the M1 macrophage polarization model with the upregulation of wound healing-related homeostatic genes further emphasizes the vital role of lactylation in the regulation of cellular function and tumor microenvironmental homeostasis [14].

Unlike previous reviews on lactylation, which primarily focused on its applications in immunotherapy [15–18], this article aims to explore the mechanisms of lactylation modification in the context of cancer. It will summarize the latest findings to provide a more comprehensive understanding of the current research landscape [19–21], delineate potential avenues for future research, and address the limitations of current cancer treatments. The integration of single-cell sequencing and spatial multi-omics will be discussed as a promising approach for overcoming these challenges. Finally, new therapeutic strategies targeting lactate-induced lactylation will be proposed.

## Key enzymes of lysine lactylation

Histones are a class of highly conserved, low-complexity proteins found in the cell nuclei of eukaryotic organisms. The DNA is wrapped around the histones to form a chain of beads, each of which is called a core nucleosome, containing an octamer (consisting of two monomers of the four core histones H2A, H2B, H3, and H4) [22]. These core histones have a C-terminal histone-fold domain and a unique N-terminal tail. The histone N-terminal tail is not significantly involved in nucleosome structure, but in interactions with other proteins and nucleosomes. Histones are modified through the addition of chemical groups to their globular structural domains and to the N-terminal tails protruding from the nucleosome core particles, with acetylation and methylation being two of the most well-studied histone marks [23]. In recent years, new forms of modifications, such as propionylation, serotoninylation, glutarylation, acetylation, etc., have been discovered [24].

**Fig. 1:**
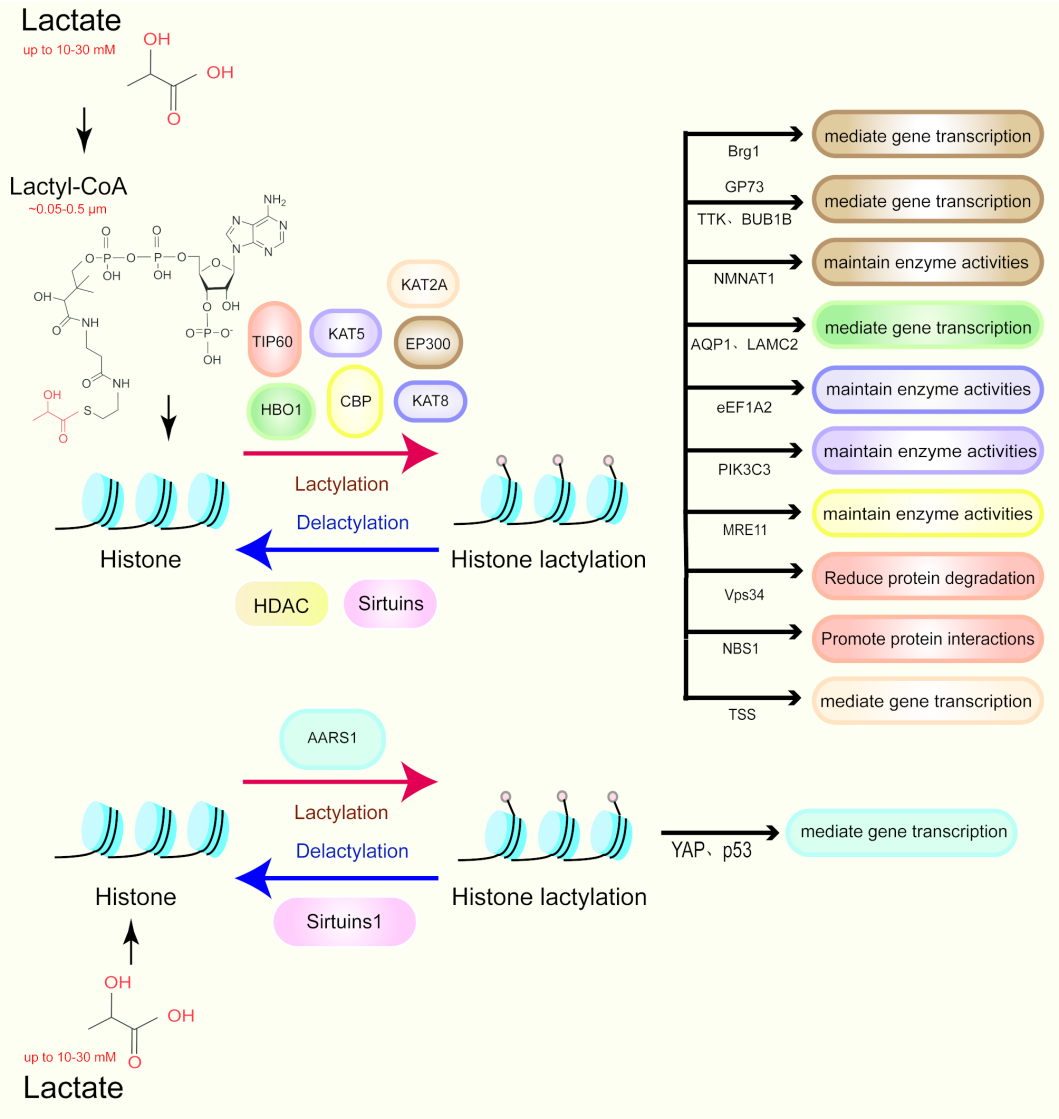
Overview of lactylation-related enzymes and substrates. **a)** EP300 acts as a transferase, regulating gene transcription mediated by GPR73, TTK, BUB1B, Brg1 and modulating the activity of NMNAT1 maintenance enzymes; **b)** HBO1 acts as a transferase, regulating gene transcription mediated by AQP1, LAMC2; **c)** KAT8 and KAT5 act as transferases, regulating eEF1A2, PIK3C3 to maintain enzyme activity; **d)** CBP acts as a transferase, modulating MRE11 to maintain enzyme activity; **e)** TIP60 acts as a transferase, regulating Vps34 to reduce protein degradation, and controlling NBS1 to promote protein synthesis; Lactate transferases with lactate as a direct substrate: AARS1 acts as a transferase, regulating YAP, p53 to mediate gene transcription

Lactylation modification refers to the accumulation of lactate to modify lysine residues on histones during cellular metabolism. This dynamic and reversible modification is regulated by lactyltransferases and delactylases, which add or remove lactoyl groups from lysine residues (Fig. 1). These modifications are recognized by effector proteins, which subsequently modulate downstream signaling pathways and biological processes [25] (Table 1).

**Table 1:** The Enzymes of Lactylation

Lactyltransferase	Substrate	Modification Site	Delactylase	Biological Function	Cancer	PMID
EP300	NMNAT1	K128	HDAC1	Enhance the nuclear localization of NMNAT1 and maintain its activity, thereby collectively contributing to the maintenance of NAD levels in the nucleus.	Pancreatic Ductal Adenocarcinoma	[26]
EP300	H3	K18	HDAC2	Promote the transcription of TTK protein kinase (TTK) and BUB1 mitotic checkpoint serine/threonine kinase B (BUB1B).	Pancreatic Ductal Adenocarcinoma	[27]
HBO1	H3	K9	——	HBO1 is highly enriched at the transcription start sites of actively transcribed genes and may directly participate in H3K9la-mediated gene regulation, thereby promoting tumorigenesis.	Cervical cancer	[31]
KAT8	eEF1A2	K408	——	eEF1A2K408la stimulates translation elongation, thereby enhancing protein synthesis in colorectal cancer cells.	Colorectal cancer	[32]
KAT5	PIK3C3	K356	——	Promote enzymatic activity and activate autophagy in muscle cells and cancer cells.	——	[33]
TIP60	NBS1	K388	HDAC3	Promote homologous recombination-driven DNA repair and chemotherapy resistance in cancer cells.	Gastric cancer	[34]
CBP	MRE11	K673	SIRT1/SIRT2	Promote DNA repair and chemotherapeutic resistance in cancer cells.	——	[35]
KAT2A	H3	K14/K18	——	Promote the expression of Wnt/β-catenin, NF-κB, and PD-L1, as well as the growth of brain tumors and immune evasion.	Glioblastoma Multiforme	[36]
AARS1	YAP/TEAD1	K90/K108	SIRT1	Activate the expression of downstream target genes to promote the proliferation of tumor cells.	Gastric cancer	[37]
AARS1	p53	K120/K139	SIRT1	Block p53 liquid-liquid phase separation, bind to DNA, and induce its target genes promotes tumorigenesis.	Breast cancer	[38]
AARS2	PDHA1/CPT2	K336/K457	SIRT3	Lactylation of mitochondrial proteins integrates interconnected metabolism and hypoxia to restrict oxidative phosphorylation.	——	[40]

In Zhang’s study, p300 was identified as a potential “writer” for lactylation. Subsequent research on lactate-induced enhancement of nicotinamide mononucleotide adenylyltransferase (NMNAT1) lactylation, which promotes pancreatic cancer cell survival under glucose deprivation, revealed that lysine 128 (K128) of NMNAT1 is a key site for lactylation. This finding confirmed that EP300 is the primary lactyltransferase catalyzing this modification, as supported by later studies [26–28]. Lysine acetyltransferases (KATs), which are involved in a variety of acylation reactions, and their main family includes GCN5-related *N*-acetyltransferase (GNAT) and MYST (MOZ, YBF2, SAS2, and TIP60), in addition to p300/CREB-binding protein (p300/CBP) [29]. The GNAT family member YiaC has been shown to function as a lactate transferase for lysine lactylation in Escherichia coli (E. coli) [30], while the MYST family member histone acetyltransferase binding to ORC1 (HBO1, also known as KAT7), as well as KAT5 and KAT8, can also catalyze lysine lactylation in mammals, acting similarly to p300 to stimulate gene expression [31–33]. In addition, lactylation of Nijmegen breakage syndrome 1 (NBS1) at lysine 388 (K388) site is critical for the formation of the MRE11-RAD50-NBS1 (MRN) complex and homologous recombination (HR) repair of DNA double-strand breaks, with TIP60 identified as the lactate transferase responsible for this modification [34]. Similarly, studies on MRE11 have shown that CBP catalyzes lactylation at lysine 673 (K673) [35]. The recently discovered lactate synthase acetyl-CoA synthetase 2 (ACSS2) translocates to the nucleus upon phosphorylation at the S267 site by extracellular signal-regulated kinase (ERK). In the nucleus, ACSS2 forms a complex with KAT2A, acting as a lactyltransferase. This complex mediates the lactylation of histone H3 at lysine 14 and lysine 18 in the promoter regions of key genes involved in signaling pathways such as Wnt and NF-κB [36].

However, it has been suggested that p300 may require lactate-coenzyme A (lactyl-CoA) as a lactyl-donor, whereas the enzyme responsible for lactyl-CoA production in mammalian cells is unknown, and levels of lactyl-CoA in tumor cells are virtually undetectable. Recently, alanyl-tRNA synthetase 1 (AARS1) was found to be a bona fide lactyltransferase that catalyzes the lactylation of proteins directly using lactate and ATP [37, 38]. The alanyl-tRNA synthetase is able to specifically catalyze lysine aminoacylation [39]. Lactate is structurally similar to alanine. After the discovery that AARS1 can act as a lactate transferase, a recent study has been shown that AARS2 can also act as a lactyltransferase to promote the lactylation of Pyruvate Dehydrogenase E1 Subunit Alpha 1 (PDHA1) and Carnitine Palmitoyltransferase 2 (CPT2) to inactivate the proteins, despite the sequence difference from AARS1. In this progress, alanine does not affect the lactylation [40].

Another important regulatory mechanism in lactate modifications is delactylation. A study of 18 enzymes from two major families of lysine delactylases in mammals found that class I histone deacetylases (HDACs) are the most potent lysine delactylases in vitro, exhibiting selectivity for cell-bound substrates [41]. Unlike class I HDACs, sirtuins primarily rely on NAD as a co-substrate. Previous studies have shown that the lactide removal activity of the Sirtuin family is less efficient than that of class I HDAC1-3. Notably, Sirtuin 3 (SIRT3) shows higher activity at the H4K16la site compared to other sirtuins, although its in vitro effect is still weaker than that of HDAC3 [42]. Given the different physiological activities as well as mechanisms of action of the two lysine deacylases in vivo, studies in both directions may identify crucial targets for inhibition of lactylation.

In addition to the low concentration of lactyl-CoA mentioned, the concentration of lactate is also worth investigating. Studies have demonstrated that elevated lactate levels (10 to 30 mM observed in cancer cells) are necessary for the formation of lactylation [20]. However, some individual studies have shown that both *in vitro and in vivo*, compared to 10 mM lactate, 20 mM lactate results in a decrease in lactylation levels in Treg cells, which may be due to cell death caused by a decrease in pH resulting from excessive lactate [43]. Besides, regarding lactate transferase research, only the latest studies on AARS1 and AARS2 have mentioned basic enzyme constants, including the dissociation constant Kd and the Michaelis constant Km.

In the latest study of HBO1, it was found that the Kd value for the binding between HBO1 and lactyl-CoA is 0.52 µM. Compared to the overexpression of HBO1 alone, the co-overexpression of the HBO1-JADE1 complex leads to an increase in Kla, but not in Kac [31]. In addition to enzyme specificity, Zhai et al. proposed a new reader site Double PHD fingers 2 (DPF2), which is more likely to recognize lactylation rather than acetylation [44]. This not only suggests that there may be acyltransferases for lactylation that are different from those for acetylation, but also indicates that existing transferases may require cofactors in vivo. These studies have enriched the mechanisms of lactylation modification, although there is still much to be explored, and more data will be needed in the future to fill these gaps.

## Histone lactylation and cancer hallmarkers

**Fig. 2:**
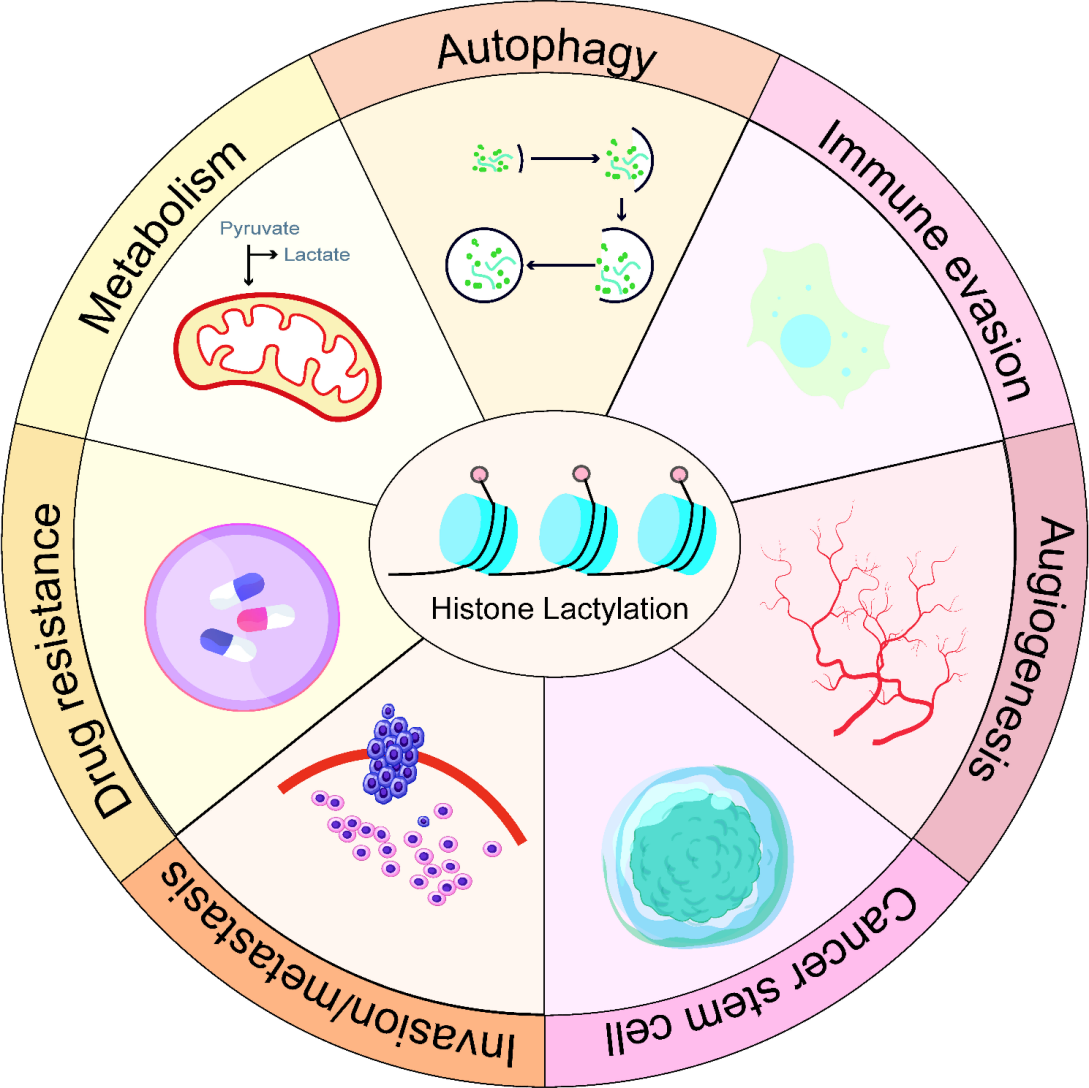
Biological functions of lactylation modification in cancer. Lactylation modification promotes tumor progression by affecting multiple hallmarks of cancer, including metabolic reprogramming, autophagy, immune evasion, angiogenesis, drug resistance, cancer stemness and tumor invasion and metastasis cancer stemness

**Fig. 3:**
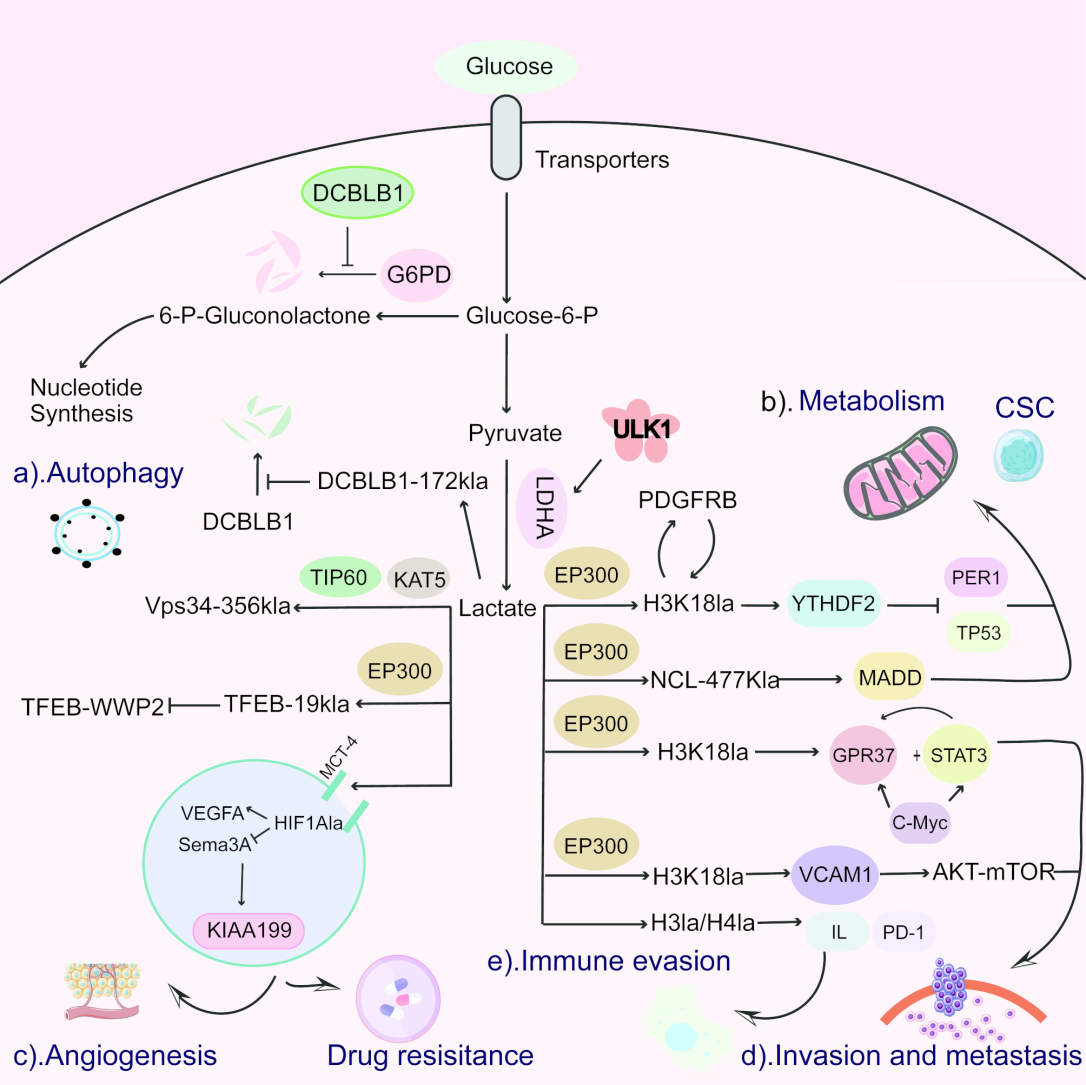
Molecular modifications of lactylation in cancer. **a) Autophagy in cells**: Lactylation of DCBLB1 can inhibit the degradation of key enzymes in the pentose phosphate pathway, thereby extending their existence, while also inhibiting its own degradation to form a positive feedback loop. In contrast, the lactylation of Vps34 and TFEB-19 increases autophagy flux. **b) Cancer metabolic reprogramming and stemness**: High glycolysis in cancer creates an environment for lactylation modifications, which can affect cancer stemness by promoting the transcription of YTHDF2, NCL, and PDGFRβ, thus facilitating the occurrence and development of tumors. **c) Angiogenesis and drug resistance**: Lactate, mediated by MCT4, induces the lactylation of HIF-1α, promoting the expression of angiogenic factor VEGFA, while inhibiting the expression of semaphorin 3 A (Sema3A), thereby driving the transcription of KIAA1199, enhancing angiogenesis, and leading to chemotherapy resistance. **d) Tumor proliferation and metastasis**: Lactylation of H3K18 activates the transcription of GPR37, promoting cancer metastasis, and in another type of tumor cell, upregulates the transcription of VCAM1, thereby activating the AKT-mTOR signaling pathway to enhance the proliferation and migration of gastric cancer cells. **e) Immune evasion**: Lactylation modification of histones regulates IL and PD-1, thereby inhibiting the antiviral activity of immune cells and promoting immune evasion of tumors

Building on the six established hallmarks identified in 2000 [45], Dr. Hanahan and Dr. Weinberg added new characteristics in 2011, including metabolic reprogramming and immune evasion [46]. As lactate, the substrate for lactylation, is a product of glycolysis and is abundant in the tumor microenvironment, it may influence cancer hallmarks such as metabolic reprogramming, resistance to cell death, immune evasion, angiogenesis, metastasis, invasion, cancer stemness, and drug resistance (Fig. 2). This article will focus on how lactylation modification drives these hallmarks (Fig. 3).

### Lactylation and cancer metabolic reprogramming

Metabolic reprogramming is a hallmark of cancer that supports rapid cell growth and proliferation by altering energy metabolism [46]. Tumor cells predominantly rely on glycolysis to generate ATP for growth, while also utilizing the pentose phosphate pathway (PPP) and the serine metabolism pathway to produce biomolecules required for cell replication [47]. In hypoxic environments, tumor cells generally produce pyruvate via the glycolytic pathway, which in turn produces lactate, rather than entering the mitochondria and being converted to acetyl-coenzyme A to produce ATP. This metabolic shift helps maintain cellular energy under low-oxygen environments. Notably, even in the presence of sufficient oxygen, tumor cells still prefer glycolysis to fulfill their energy requirements, which is known as aerobic glycolysis [13].

Ocular melanoma exhibits increased glycolysis, which produces higher levels of lactate than normal tissues. This excess lactate serves as a substrate for lactylation. Specifically, H3K18 lactylation (H3K18la) has been shown to activate the transcription of YTH N6-methyladenosine RNA-binding protein F2 (YTHDF2), which recognizes m6A modifications on tumor suppressor genes such as period circadian regulator 1 (PER1) and tumor protein p53 (TP53), promoting their degradation. Moreover, higher levels of lactylation indicate early recurrence and increased invasiveness of cancer [48]. Similarly, in clear cell renal cell carcinoma (ccRCC), which is characterized by high levels of glycolysis, inactivation of von Hippel-Lindau (VHL) protein induces histone lactylation. H3K18la clusters predominantly around the platelet-derived growth factor receptor β (PDGFRβ) and activates its transcription, contributing to the poor prognosis of ccRCC [49]. Additionally, a recent study in intrahepatic cholangiocarcinoma (ICCA) showed that increased glycolysis in ICCA cells leads to enhanced lactylation of nucleolin, further promoting the malignant phenotype of these cells [50]. Analysis of data from The Cancer Genome Atlas (TCGA) revealed that Rho GTPase Rif (RHOF) is overexpressed in pancreatic cancer. Further research showed that RHOF promotes Pyruvate kinase 2 (PKM2) expression by upregulating c-Myc, thereby enhancing the glycolysis of tumor cells. This, in turn, leads to the lactylation of Snail1, which promotes the migration, invasion, and epithelial-mesenchymal transition (EMT) of pancreatic cancer [51]. Similar findings has also been reported in gastric cancer [52], glioma [53], and lung adenocarcinoma [54], breast cancer [55].

The glycolytic activity associated with tumor metabolism creates favorable conditions for lactylation modification. In turn, lactylation modification has been shown to enhance metabolic reprogramming in various cancers, including lung cancer [56], hepatocellular carcinoma (HCC) [57, 58], and cervical cancer [59]. A global lactylome analysis of a HCC cohort revealed that lactylation could preferentially affect enzymes involved in metabolism pathways [57]. In cervical cancer, lactylation of Discoidin, CUB, and LCCL domain-containing type I (DCBLD1) has been shown to prolong the activity of the PPP [59]. Additionally, lactylation promotes glycolysis in endometrial cancer cells by activating the phosphatidylinositol 3-kinase (PI3K)/Akt pathway and the hypoxia-inducible transcription factor (HIF) pathway [60]. These findings suggest that lactylation acts as a “bridge” between metabolic reprogramming and cancer progression. However, whether targeting lactylation can lead to more effective therapeutic outcomes remains to be validated by further clinical studies.

### Lactylation and resistance to cell death

Resistance to cell death is another significant hallmark of cancer. Autophagy, a striking process, mainly consists of upstream regulators and downstream effector components [61]. In the PPP, DCBLD1 is capable of inhibiting the autophagic degradation of glucose-6-phosphate dehydrogenase (G6PD), thereby enhancing PPP activity to promote cell proliferation [59]. DCBLD1 lactylation can inhibit its degradation, which further clarifies how this modification can impact cancer development by reducing autophagic degradation of key enzymes.

However, there is now also a lot of evidence revealing the bidirectional effect of autophagy in tumors, not only the traditional idea that tumor development can be inhibited through autophagy [62], but also the role of autophagy in promoting cancer, regulating tumor metabolism, and cancer metastasis [63]. The UNC-51-like kinase (ULK1) complex acts as an upstream regulator to mediate the phosphorylation of the serine 196 site of lactate dehydrogenase (LDHA) to promote lactate production. The generated lactate undergoes Vacuolar sorting protein 34 (Vps34) lactylation mediated by the acyltransferase KAT5/TIP60. This process promotes autophagic flux and endolysosomal transport [64]. In addition, lactate-induced lactylation of transcription factor EB (TFEB) at the K91 site prevents its degradation by E3 ubiquitin ligase WWP2-mediated ubiquitination, thereby increasing lysosomal activity and autophagic flux [65]. These findings suggest that lactylation modifications can either suppress or enhance autophagy, thereby facilitating the progression of tumors.

### Lactylation and immune evasion

As early as 2019, Zhang et al. discovered that lactylation modification is associated with macrophages [14]. In glioblastoma, monocyte-derived macrophages and microglia account for approximately 50% of the cellular population. The lactate produced by these cells can induce histone lactylation, which may subsequently affect the immunological microenvironment of tumor cells through the transcriptional program of interferons [66, 67]. Similarly, in gliomas, it has been found that hypoxia-mediated accumulation of lactate is taken up by macrophages, and then regulates tumor necrosis factor superfamily member 9 (TNFSF9) expression through MCT-1/H3K18La signaling, inducing M2 macrophage polarization, thereby promoting malignant progression [68]. Additionally, lactate induces histone H3K18 lactylation, which amplifies the activity of the promoters of CD39, CD73, and CCR8 genes, thereby disrupting the immune microenvironment [69].

Lactylation activates tumor immune suppression through different pathways in various cancer cell types. In head and neck squamous cell carcinoma, interleukin-11 (IL-11) was identified as a downstream target of histone lactylation at lysine 9 of histone H3 (H3K9la). Further validation showed that overexpressed IL-11 inhibits CD8 + T cell proliferation and promotes tumor development via the Janus kinase 2/signal transducer and activator of transcription 3 (JAK2/STAT3) pathway [70]. Moreover, lactate-mediated lactylation of MOESIN at Lys72 enhances TGF-β signaling through TGF-βRI, suppressing anti-tumor inflammation and promoting hepatocellular carcinoma growth [43]. In non-small cell lung cancer (NSCLC), H3K18 lactylation promotes immune suppression by inducing pore membrane protein 121 (POM121), which increases MYC activity and programmed cell death-ligand 1 (PD-L1) expression [71]. In ovarian cancer, lactate induces CCL18 expression via H3K18 lactylation in macrophages, facilitating tumor progression [72]. Additionally, in gastric cancer, lysyl oxidase (LOX) secreted by cancer-associated fibroblasts (CAFs) activates the TGFβ/IGF1 signaling pathway, augmenting glycolysis and resulting in lactate accumulation. This enriches H3K18la near the PD-L1 promoter, contributing to malignant progression [73]. In hematological malignancies such as acute myeloid leukemia, immunotherapy plays a significant role. Research has shown that the transcription factor STAT5 promotes the transcription of glycolytic enzymes such as HK1, PFKP, and PDHA. Upregulated glycolysis leads to lactate accumulation, resulting in significant enrichment of H4K5la at the PD-L1 locus, thereby increasing PD-L1 expression [74].

Lactylation modification in tumors can assist cells in suppressing immune regulation. This presents many unknown challenges for subsequent treatments.

### Lactylation and angiogenesis

Vasculogenic mimicry (VM) is a new model of tumor microcirculation. Unlike endothelial cell-dependent angiogenesis, VM is independent of endothelial cells and can provide sufficient blood supply for tumor growth [75]. In recent years, VM has been reported in a variety of malignant tumors, including melanoma, glioblastoma, osteosarcoma, HCC, prostate cancer (PCa), and colorectal cancer [76–80], and is associated with invasion, metastasis, and poor prognosis of malignant tumors [81].

Lactylation modifications contribute to neovascularization [82, 83] and promote tumor progression by regulating factors involved in angiogenesis. For example, in PCa, MCT4-mediated lactate promotes the expression of the vascular endothelial growth factor A (VEGFA) by inducing lactylation of HIF-1α and suppresses the expression of semaphorin 3 A (Sema3A), which supports the transcription of Cell migration-inducing protein (CEMIP, KIAA1199) and enhances angiogenesis [84]. In lung adenocarcinoma, elevated levels of lactylation of Solute Carrier Family 25 Member 29 (SLC25A29) limit its transcription, and low expression of SLC25A29 is associated with angiogenesis in endothelial cells [85]. In addition, the pro-angiogenic effect of Golgi protein 73 (GP73) in HCC is associated with lactylation modification, which is responsible for the upregulation of GP73 expression [86]. These findings provide new insights into the mechanisms of tumor angiogenesis and suggest potential therapeutic targets to inhibit tumor progression, alleviate patient suffering, and optimize healthcare resource utilization.

### Lactylation and tumor invasion and metastasis

Lactylation modification plays a critical role in tumor invasion and metastasis, key hallmarks of malignancy and major contributors to cancer-related mortality. Numerous studies have highlighted the significant impact of lactylation in these processes. For example, G protein-coupled receptor 37 (GPR37) enhances glycolysis and histone lactylation through the Hippo pathway, promoting the liver metastasis of colorectal cancer [87]. In esophageal squamous cell carcinoma, LAMC2 has been shown to drive the development of a malignant phenotype, with regulation by H3K9la [88]. In gastric cancer cells, H3K18 lactylation upregulates Vascular cell adhesion molecule-1 (VCAM1) transcription, activating AKT-mTOR signaling to enhance cell proliferation and migration [89]. Additionally, inhibition of H3K18la in endometrial cancer has been found to effectively hinder the proliferation and migration of cancer cells, induce apoptosis, and prevent the progression of the cell cycle from G0/G1 to S phase [60]. In HCC, inhibition of histone lactylation reduces the malignant phenotype of cancer cells in vitro and suppresses tumorigenesis and metastasis in vivo [90]. In a cohort analysis of hepatitis B virus-associated HCC, meanwhile, lactylation modification at the K28 site inhibits the function of adenylate kinase 2 and accelerates the proliferation and metastasis of HCC [57].

Current research primarily focuses on how lactylation promotes tumor invasion and metastasis by modulating downstream regulatory factors post-transcriptionally. However, other mechanisms are also emerging. For example, in breast cancer cells, Potassium Two Pore Domain Channel Subfamily K Member 1 (KCNK1) supports the lactylation of H3K18 through LDHA. This not only affects downstream regulatory factors but also stimulates the expression of LDHA, creating a positive feedback loop that further drives the development of breast cancer cells [91]. Additionally, studies have begun to explore the role of lactylation in tumor proliferation and metastasis through macrophage polarization and other immune-related mechanisms [92].

In tumor control, while management of the primary lesion is essential, subsequent tumor invasion and metastasis have become major research challenges. Tumor metastasis is usually closely associated with poor cancer prognosis, and lactylation plays an indispensable role in this process. However, many of the underlying mechanisms are not directly regulated by lactylation, and other factors involved in metastasis still require further investigation.

### Lactylation and cancer stemness

As ongoing research delves deeper into the intricacies of oncology, it has been revealed that the heterogeneity of cancer cells is a pivotal factor influencing the rate of tumor growth, invasiveness, drug sensitivity, and ultimately, the prognosis and therapeutic outcomes. The main mechanisms include genetic evolution of cancer cells, cancer stem cells, and ecological niche heterogeneity [93].

Cancer stem cells (CSCs), cell cluster with high tumorigenicity and self-renewal properties, have been found in a variety of cancers [94, 95]. Compared with other cells in the tumor, CSCs adapt to environmental changes more efficiently and show greater resistance to conventional therapies [96]. Given the properties of CSCs, lactylation in cancer may further alter cancer development and therapeutic efficacy by maintaining the stemness of CSCs. It is well known that liver cancer stem cells (LCSCs) play a significant role in tumor development and therapeutic resistance, while H3K56la, through the regulation of target genes, plays a vital role in the oncogenesis and maintenance of stemness in LCSCs. Altering the level of lactylation affects the proliferative capacity and stem cell properties of LCSCs [97]. Besides, PTBP1 lactylation promotes the maintenance of glioma stem cells through PFKFB4-driven glycolysis [98]. Additionally, in NSCLC [99], colorectal cancer [100], esophageal cancer [101], and other studies, hypoxia-induced lactylation of different molecules has a significant effect on the stemness of cancer cells, but these studies mainly focused on the regulation of cancer cell stemness by specific molecules without delving into the specific mechanisms of lactylation.

Furthermore, recent studies have shown that high expression of LncRNA can increase lactylation levels in endometriosis [102] and is also associated with the regulation of CSCs phenotype [103]. This has led to further investigation into whether IncRNA-regulated lactylation influences CSC stemness in cancer. Therefore, subsequent studies should focus on the relationship between the lactylation of proteins and the maintenance of cancer cell stemness in order to better understand the mechanisms of cancer progression and drug resistance.

### Lactylation and drug resistance

In cancer treatment, drug resistance presents a significant challenge over the long-term therapeutic process. Once tumor cells develop resistance to chemotherapeutic agents, the effectiveness of treatment diminishes. The study of lactylation may be possible to find complementary drugs to the currently available therapeutic regimens. For example, bevacizumab plays a critical role in both first- and second-line treatment for metastatic colorectal cancer, but most patients eventually develop intrinsic or acquired resistance. Existing research indicates aerobic glycolysis-induced high histone lactylation levels enhance Rubicon Like Autophagy Enhancer (RUBCNL) transcription, which stimulates autophagy by promoting autophagosome maturation to further aggravate colorectal cancer. Upon receiving bevacizumab treatment, the glycolysis in hypoxic cancer cells may be further enhanced, subsequently leading to increased levels of histone lactylation and the transcriptional upregulation of RUBCNL through lactylation, which contributes to the survival of colorectal cancer cells and resistance to treatment. Hence, inhibition of histone lactylation may be a novel strategy to improve the efficacy of bevacizumab [104].

Additionally, vascular inhibitors are a primary strategy in targeted therapy for solid tumors [105]. However, these angiogenesis inhibitors can cause extensive vascular collapse, leading to tumor hypoxia, which activates HIF1α, triggering glycolysis and lactate secretion in hypoxic regions of the tumor [106]. The accumulation of extracellular lactate induces MCT1-mediated lactate uptake in normoxic regions, where it contributes to histone lactylation [84]. Therefore, lactate inhibitors may be used in combination with vascular inhibitors to enhance therapeutic efficacy by disrupting this metabolic feedback loop.

In addition to the drug resistance associated with lactylation due to increased glycolysis, lactylation plays a critical role in resistance mechanisms across various cancers. In recurrent glioblastoma, H3K9la is significantly enriched at the LUC7L2 promoter, activating its transcription and inhibiting mismatch repair, which ultimately contributes to drug resistance [107]. Similarly, in glioma, lactylation of X-ray cross complementing protein 1 (XRCC1) has been linked to DNA repair mechanisms that generates chemotherapy resistance [108]. In bladder cancer, H3K18la upregulates the expression of the transcription factor Yin Yang 1 (YY1) and the Y-box binding protein 1 (YBX1), promoting resistance to cisplatin [109]. Besides, in a resistance model of Lenvatinib, a multi-targeted receptor tyrosine kinase inhibitor used as a first-line therapeutic agent for advanced HCC, lactylation of the m^6^A-reader IGF2BP3 mediates the synthesis of serine and S-adenosylmethionine, which further upregulates phosphoenolpyruvate carboxykinase (PCK2). This forms a positive feedback loop that enhances antioxidant capacity, thereby conferring cellular resistance to Lenvatinib [110]. These findings suggest that lactylation modifications can directly induce drug resistance, and targeting lactylation pathways with inhibitors could potentially improve the sensitivity of tumors to existing therapies. Lactate also promotes DNA damage repair in tumors by the lactylation of the NBS1 protein, contributing to chemoresistance. Notably, there is strong synergy between the clinically available LDHA inhibitor stiripentol and genotoxic therapies, such as cisplatin and ionizing radiation (IR), which can reduce tumor resistance to these treatments [34]. LDHA inhibitors are currently the more commonly mentioned and well-documented lactate-modified inhibitors in the clinical setting and are mainly used as complementary agents.

Lactylation regulates critical tumor processes, including metabolic reprogramming, oncogenesis, autophagy, immunosuppression, angiogenesis, and chemotherapy resistance. Targeting lactate production may provide a promising strategy for cancer treatment.

## Lactylation and clinical applications

### Lactylation as biomarkers

Previous researches have demonstrated the role of lactylation modification in various stages of tumor development. For instance, the relative density of H3K18la has been identified as a potential diagnostic marker for septic shock, exhibiting high sensitivity and specificity. This suggests that lactylation modification could serve as valuable biomarkers for tumors. Since most tumors are diagnosed at advanced stages, lactylation may provide an opportunity for early detection. Additionally, genes such as KCNK1 [91], SHMT2 [101], and PYCR1 [111], which influence tumor metastasis and invasion through histone lactylation, may also serve as biomarkers to help track tumor progression.

Lactylation exists in gastric cancer [112], lung cancer [64], colorectal [113], cancerhepatocellular carcinoma [110], and other cancers. In gastric cancer, lactylation expression is significantly higher in tumor tissues compared to adjacent normal tissues [112]. Similar findings have been reported in other cancers, such as lung cancer [64] and pancreatic cancer [26], where elevated lactylation levels correlate with cancer progression and differentiation. A study in colorectal cancer demonstrated that patients with high lactate levels had higher recurrence rates, more distant metastasis, and more advanced cancer stages [113].

In gastric cancer, Yang et al. identified six lactate-related genes linked to cancer development through cluster analysis and principal component analysis (PCA). Their lactate score model revealed that higher lactylation scores were associated with increased tumor proliferation, metastasis, and invasion [114]. Kaplan-Meier analysis further confirmed that high lactylation levels are correlated with poorer overall survival in gastric [112] and pancreatic cancer patients [26]. Similarly, patients with high lactylation levels of NBS1 had a much lower survival rate than patients with low levels of NBS1 [34]. In the Shanghai Tongji Hospital cohort, high IGF2BP3lac levels are associated with poorer progression-free survival and overall survival in HCC [110].

The lactylation-related genes mentioned earlier have been extensively studied in predicting the prognosis of cancer. The current international grading system for multiple myeloma is based on albumin and β2-microglobulin. Considering its current limitations, the latest study, after extracting the expression profiles of lactylation-modified related genes (LRGs), found that 16 genes are associated with the prognosis of multiple myeloma after applying one-way Cox regression and constructed a prediction model after further multifactorial Cox regression and LASSO regression to build the characteristic risk scores of LRGs. After validation, it was found that there are differences between the high-risk group and the low-risk group in terms of chemotherapy resistance, immune infiltration, and mortality outcomes [115]. In another study, 99 patients with triple-negative breast cancer were followed up and found that the risk of death in patients with high H4K12la expression is much higher than that in patients with low or negative H4K12la expression [116]. Besides, lactylation scores in HCC are associated with immunotherapy, chemotherapy resistance, and tumor progression [117]. The gene score has also been shown to be associated with immunotherapy resistance and tumor progression in ovarian cancer [118], ccRCC [119], and pancreatic ductal adenocarcinoma [120]. Advancing our understanding of lactylation-related genes has the potential to lead to the development of more accurate prognostic models for cancer. However, a pan-cancer analysis across 33 types of cancer revealed that the prognostic evaluation results of lactylation scores are associated with tumor types [121]. The lack of population-based assays and limited clinical evidence on lactylation as an early biomarker highlight the need for additional clinical studies to clarify its role and validate its utility in cancer diagnosis and treatment.

### Lactylation and therapeutic approaches

**Fig. 4:**
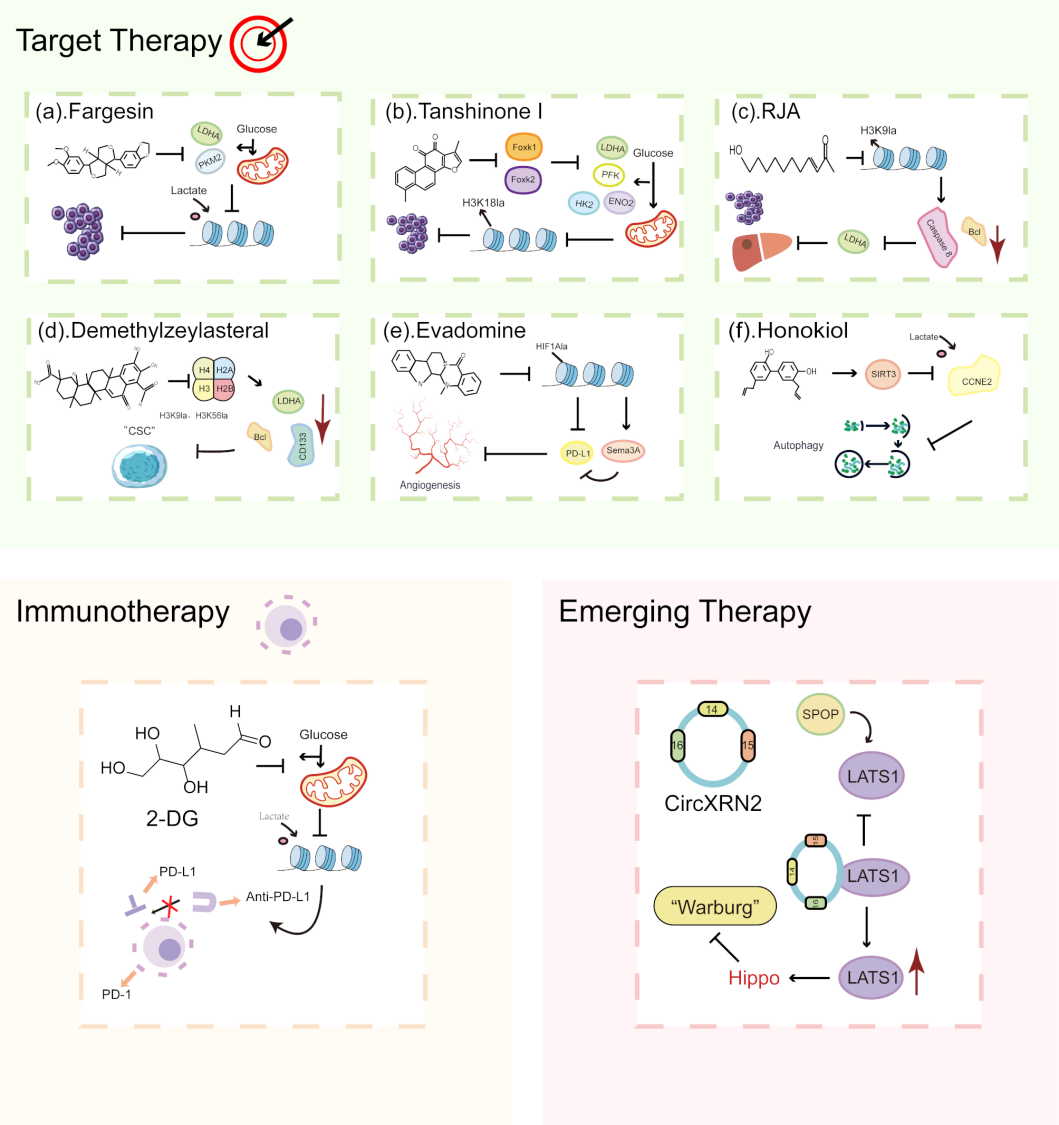
Anticancer strategies targeting lactylation. **1. Target therapy** **a) Fargesin**: By targeting key enzymes PKM2 and LDHA in the glycolytic process, it inhibits histone lactylation, reducing cell proliferation. **b) Tanshinone I**: By inhibiting Foxk1/Foxk2, the expression of glycolysis-related enzymes, such as LDHA, PFK, HK2 and ENO2, is reduced. This inhibition of H3K18la slows down the progression of cancer. **c) RJA**: By inhibiting H3K9la, it reduces the expression of Caspase 8 and Bcl, thereby inhibiting LDHA and suppressing the development of hepatocellular carcinoma. **d) Demethylzeykasteral**: By inhibiting H3K9la and H3K56la, it reduces the expression of Bcl, LDHA, and CD133 proteins, inhibiting cancer stemness. **e) Evadomine**: It can inhibit HIF-1α lactylation on histones, suppress the expression of PD-L1, and promote Sema3A to inhibit angiogenesis. **f) Honokiol**: Targets SIRT3 to inhibit the lactylation of CCNE2, suppressing cell autophagy. **2. Immunotherapy** **2-DG** (as an example): Glycolysis inhibitors reduce the production of lactate, thereby inhibiting histone lactylation and consequently enhancing the efficacy of anti-PD-L1 therapy. **3. Emerging therapy** **CircXRN2**: After binding with LATS1, it inhibits the ubiquitination of LATS1, increases LATS1 levels, activates the Hippo pathway, and suppresses the Warburg effect in cancer, thereby affecting cancer progression

There is now a consensus on the impact of lactylation in cancer, and therapeutic agents targeting its targets are constantly being investigated. Currently, strategies targeting lactylation modification are predominantly composed of targeted therapies (Fig. 4), immune combinational treatments, and novel therapeutic approaches.

The mechanisms underlying lactylation primarily encompass lactate derived from glycolysis serving as a substrate, lactyltransferases, delactylases, and specific lactylation modification sites. Correspondingly, the drugs utilized in targeted therapies predominantly consist of glycolysis inhibitors, delactylase activators, and agents that specifically target lactylation sites. Glycolysis can provide substrates and create a suitable environment for lactylation in cancer cells, and targeting lactate production inhibited by glycolysis has been shown to be effective in bladder cancer [109] and colorectal cancer [104]. 2-Deoxy-D-glucose (2-DG), a synthetic glucose analog, accumulates within cells to inhibit glycolysis and glucose metabolism [122]. In lung cancer [99] and endometrial cancer [60], 2-DG effectively reduces lactylation by lowering lactate production. Although many clinical studies have demonstrated its combined anticancer effects, 2-DG’s rapid metabolism and short half-life limit its therapeutic potential [123]. In contrast, a new lignan, fargesin, which was isolated from the magnolia plant, inhibits tumor growth by targeting pyruvate kinase 2 (PKM2), the rate-limiting enzyme in glycolysis. It also suppresses histone H3 lactylation, positioning it as a promising candidate for cancer therapy [124]. Additionally, in glioblastoma research, dexmedetomidine was found to reduce c-Myc lactylation by inhibiting glycolysis, thereby affecting cancer cell metastasis. However, the lack of animal models restricts its use to preclinical exploration, offering new targets for future treatments [125]. Research has shown that Tanshinone I significantly affects specific histone modifications, particularly reducing acetylated histone H3 at lysine 18. Additionally, it can help alleviate the immunosuppressive tumor microenvironment [126]. Currently, research on glycolysis inhibitors is extensive, with studies exploring their combination with existing therapeutic strategies, including resistance to therapies [127, 128], immunotherapy [129–131], and other emerging approaches. However, as a monotherapy, glycolysis inhibitors have yet to produce definitive results, highlighting the limitations of their non-targeted application.

Targeting lactylation has shown promise as an effective strategy in cancer treatment. For instance, the natural compound Royal Jelly Acid (RJA) demonstrates significant antitumor effects both *in vitro and in vivo*. It inhibits the proliferation and migration of HCC while promoting apoptosis. Mechanistically, RJA reduces histone lactylation at the H3K9la and H3K14la sites, interfering with lactate production. Notably, the level of lactylation correlates with the tumor proliferation marker Ki67, suggesting that RJA may impede HCC progression. In a subcutaneous tumor model in nude mice, RJA is also effective in suppressing tumor growth, highlighting its potential as a therapeutic agent for HCC by targeting H3 histone lactylation [132].

Similarly, demethylzeylasteral (DML), a triterpenoid antitumor compound, has been shown to inhibit the proliferation, migration and induce apoptosis in LCSCs. DML works by inhibiting histone lactylation at the H3K9la and H3K56la loci, thereby regulating glycolysis in a manner similar to RJA. In a tumor xenograft model in nude mice, it was confirmed that the anti-hepatocellular carcinoma effect of DML is mediated by regulating H3 lactylation, showing its promise as a potential therapeutic agent for HCC [133]. Another compound, Evodiamine, has been studied in a PC-3 cell xenograft model in nude mice, where it was found to inhibit tumor growth. Evodiamine exerts its effects by suppressing Sema3A, a key regulator of angiogenesis, and reducing programmed death receptor-1 (PD-1) expression through inhibition of HIF1α lactylation at the H3K18 site. Furthermore, Evodiamine induces ferroptosis in PCa cells, highlighting its potential as a therapeutic agent targeting lactylation in PCa [134].

The roles of lactate transferase and delactonase are particularly important in the process of lactylation modification. SIRT3, which has been shown to exhibit delactonase activity [42]. In a mouse model of HCC, activation of SIRT3 with the compound Honokiol (HKL) altered the lactylation of E-type cyclins CCNE2, induced apoptosis in cancer cells, and reduced liver damage while inhibiting tumor proliferation [135]. These findings suggest that delactonase activators, such as HKL, hold promise as novel therapeutic agents for inhibiting tumor proliferation and enhancing cancer treatment outcomes.

Several drugs mentioned in research have not been explicitly evaluated for their toxicity or the required dosages. Literature has indicated that evodiamine exhibits hepatotoxicity [136], yet no specific studies have established a dose-response relationship for the toxicity induction. Moreover, honokiol has been proven to have certain effects in anti-inflammatory, antibacterial, and anticancer activities, and it also possesses hepatoprotective properties. However, it has been observed to cause reproductive damage to the testes in male rats [137].

Immunotherapy has significant potential in cancer treatment, yet research indicates that lactate-driven lactylation can impede its efficacy [69, 73]. Combining glycolysis inhibitors with immunotherapy may offer a solution. For instance, in NSCLC, oxalate combined with anti-PD-1 therapy effectively suppresses Ki67 + tumor cells [71]. In glioblastoma, the oxalate-CAR-T combination extends survival in tumor-bearing mice compared to CAR-T monotherapy [69]. In HCC, where immunotherapy is less effective [58], reducing lactate levels alongside anti-PD-1 treatment enhances anti-tumor responses [43]. The latest research on serine diet has revealed that it not only enhances antitumor immune responses but also stimulates lactylation modification, which promotes immune evasion in tumors. This finding implies that combining serine diet with immunotherapy may potentially reduce the occurrence of adverse effects [138]. These findings pave the way for overcoming immunotherapy’s limitations.

Additionally, there are several innovative therapeutic approaches. With the rapid advancement of high-throughput sequencing and molecular biology, non-coding RNAs, particularly circular RNAs (circRNAs), have emerged as key regulators of various physiological and pathological processes, including cancer development [139]. Recent studies have demonstrated that circRNAs are not only negative regulators of glycolysis and lactate production, but also inhibit histone lactylation through activation of the Hippo pathway, thereby preventing further tumor progression [140]. Notably, the Hippo pathway itself is activated to suppress histone lactylation, which in turn impedes tumor development. This suggests the potential for developing small molecule drugs targeting CicrXRN2. Cold atmospheric plasma (CAP) has been documented to suppress the activity of LDHA, emerging as a novel multi-modal approach in tumor treatment [141]. This therapy stands out for its minimal side effects, ability to curb metastasis, and manageable drug resistance, giving it an edge over conventional tumor therapies. Regrettably, further exploration of this therapeutic avenue has seemingly stalled. Nonetheless, targeting lactylation or integrating it with CAP holds promise for delivering the anticipated therapeutic outcomes.

While much research has been conducted on targeting glycolysis for cancer treatment, with compounds like fargesin targeting PKM2 and phosphofructokinase (PFK-1) knockdown shown to inhibit bladder cancer progression [142], clinical data remains insufficient to fully elucidate the mechanisms of these drugs (Table 2). Moreover, the high similarity between the acetylation sites and lactylation sites may pose a potential threat of off - target effects [143]. In the future, combination therapy could provide a breakthrough for this issue. However, the potential for increased toxicity from combination drugs must also be evaluated through more animal models and clinical data.

**Table 2:** Existing lactylation-related drugs

Cancers	Drugs	Trial	Outcomes	Adverse effect	PMID
Soild cancer	2-DG	Phase I/II	2DG at 63 mg/kg in combination with weekly docetaxel was well-tolerated in this study and is the recommended phase II dose for daily 2DG administrationr	Fatigue, Sweating, Dizziness and Nausea	[123]
NSCLC	Fargesin	In vitro/In vivo	Targeting PKM2 to disrupt histone H3 lactylation inhibits tumorigenesis in NSCLC.	——	[124]
Glioblastoma Multiforme	Dexmedetomidine	In vitro	Inhibit the lactylation of c-Myc reduces protein stability, thereby suppressing the migration, invasion, and glycolysis of GBM cells.	Hypertension, Hypotension, or Bradycardia	[125]
Ovarian cancer	Tanshinone I	In vitro/In vivo	Inhibition of lactate production reduces the level of H3K18la, thereby decreasing the expression of tumor-associated genes.	——	[126]
HCC	Royal jelly acid	In vitro/In vivo	Disrupt lactate production and inhibit the lactylation of H3K9la and H3K14la sites to suppress the development of HCC.	——	[132]
HCC	Demethylzeylasteral	In vitro/In vivo	Inhibiti the lactylation of histone H3 to suppress the tumorigenicity induced by liver cancer stem cells.	Potential hepatotoxicity	[133]
Prostate cancer	Evodiamine	In vitro/In vivo	Restricting histone lactylation and the expression of HIF1A in PCa cells significantly blocks lactate-induced angiogenesis.	Hepatotoxicity	[134]
HCC	Honokiol	Phase I (NSCLC)	Activating SIRT3 induces apoptosis in HCC by regulating the level of Kla on CCNE2.	Hepatotoxicity	[135]

## Lactylation and Single-cell sequencing and Multi-omics analyses

In the realm of contemporary research, single-cell sequencing and multi-omics analyses have emerged as pivotal tools, garnering extensive attention across a multitude of scholarly publications. Specifically, in the study of oral squamous cell carcinoma, enrichment analysis has proven instrumental in elucidating the pathways modulated by lactylation modifications. Moreover, proteomic lactylation sequencing data have enabled the precise identification of specific lactylation sites, thereby enhancing our understanding of these modifications at a molecular level [144].

The advent of single-cell sequencing technology has revolutionized our approach to deciphering the intricate nature of tumor heterogeneity. By capturing the entire transcriptome at single-cell resolution, this technology facilitates the differentiation of various cell types within complex tumor tissues. Complementing this, spatial transcriptomics addresses the spatial constraints inherent in single-cell sequencing, providing a more comprehensive view of cellular interactions and tissue architecture. The synergistic application of these advanced techniques has yielded significant insights, particularly in hepatocellular carcinoma. Single-cell sequencing technology has revealed the existence of heterogeneity between HSC1 and HSC2 cells in HCC. To further clarify the spatial distribution of cell subpopulations in the HCC tumor microenvironment, spatial transcriptomics using Cell2location was conducted. It was found that HSC1 and HSC2 have distinct spatial distribution patterns compared to other non-tumor cell subpopulations, indicating their spatial heterogeneity in HCC. Based on this, the main pathways of HSC cells were explored to identify potential lactylation targets such as AKR1B10 [145]. Single-cell sequencing technology also discovered that overexpression of GP73 in HCC cells activates angiogenesis, and spatial transcriptomics supplemented this by showing that HCC cells typically account for a high proportion among all cell subclusters [86].

In clear cell renal cell carcinoma, single-cell sequencing identified histone modifications in mesangial cells, and spatial transcriptomics confirmed that cancer-associated fibroblasts are spatially closely connected to cancer cells [119]. Similar applications have also found that lactylation-modified cell clusters have characteristics of high glycolysis and low embryonic diapause decline feature scores [146]. The combination of these technologies brings a deep analysis of tumor heterogeneity, laying the foundation for finding precise targets of action.

Leveraging these foundational advancements, researchers are now better equipped to predict biomarkers and establish robust prognostic models, thereby advancing the frontiers of cancer diagnostics and therapeutics [117]. This integrative and multidimensional approach to cancer research not only enhances our mechanistic understanding but also paves the way for more personalized and effective treatment strategies.

## Conclusions and perspectives

In conclusion, lactylation modification, an emerging epigenetic modification, primarily involves the modification of target molecules by lactate produced during glycolysis, which subsequently influences downstream regulatory pathways. This modification plays a key role in metabolic reprogramming in cancer, acting as a positive feedback loop that drives tumor progression. It also has a dual impact on cellular autophagy, promoting tumor development, enhancing cancer cell stemness, and increasing chemoresistance.

Despite our initial understanding of the mechanisms of lactylation modification in tumorigenesis and progression, there are still significant challenges in utilizing this knowledge to develop more effective clinical therapeutic regimens. Firstly, due to the heterogeneity of tumors, lactylation modification can exhibit different mechanisms of action, and even produce opposite effects. For example, in cervical cancer, lactylation modification can inhibit the degradation of DCBLD1, thereby inhibiting the degradation of the G6PD rate-limiting enzyme, thus prolonging the existence of PPP and promoting cervical cancer [59]. However, in the mechanism of cervical cancer caused by human papillomavirus 16 E6, it was found that this virus inhibits the lactylation of G6PD, promoting the formation of G6PD dimers and activating the pentose phosphate pathway to promote the malignant transformation of tumors [147]. The development of single-cell sequencing and multi-omics technologies can solve this problem to a certain extent. By classifying cells according to their characteristics, a better explanation of the cellular environment on which lactylation mechanisms depend can be provided.

Secondly, in the process of summarizing current research, it was found that lactylation modification-related enzymes, sites of action, and required cellular environments are highly similar to many epigenetic modifications. For example, in the proteomics analysis of hepatocytes, it was found that as a member of the deubiquitinase family, the deubiquitination activity of USP14 in hepatocellular carcinoma maintains the stability of HIF1-α [148]. At the same time, the lactylation level of lysine at the 336th amino acid site of USP14 in HCC was abnormally elevated, indicating that there is a cross-interaction between lactylation and ubiquitination. Apart from the possible cross-interaction, in esophageal cancer, it was found that hypoxia treatment-induced lactylation of Axin1 protein promotes the ubiquitination modification of Axin1 protein, promoting glycolysis and cell stemness of esophageal cancer cells [149]. In ocular melanoma, it was found that histone lactylation enhanced the expression of ALKBH3, while weakening the existence of tumor suppressor promyelocytic leukemia protein (PML) aggregates, and promoting the m1A methylation of SP100A, promoting the malignant transformation of cancer [150]. These all indicate that lactylation modification may have synergistic effects with other modifications. Compared with other epigenetic modifications, acetylation and lactylation modification have a high degree of similarity in metabolic pathways, writer enzymes, de-lactylases, and even target sites of action. Therefore, it is necessary to consider the competitive relationship between the two in different tumor cells or different types of cells. For example, In tumor-associated macrophages isolated from B16F10 melanoma and LLC1 lung tumors, the expression of Arg1 is positively correlated with the level of histone lactylation, but not with the level of histone acetylation [14]. However, the kinetics of histone lactylation at lysine sites (24 h) is slower than that of histone acetylation at lysine sites (6 h), indicating that under physiological conditions, acetylation is favored over lactylation. Whether malignant cells have altered the kinetics of histone lactylation to make it competitively superior to acetylation is still another interesting question and a potential target opportunity worth exploring [141].

Finally, the cross-interaction of epigenetic modifications brings up a serious issue: whether existing targeted drugs might actually go off-target during their actual effects, which could reduce the efficacy of current drugs. In addition to this, there are gaps in the research on drug dose-response relationships, pharmacokinetics, and toxic side effects. However, the latest studies show that low-dose SIRT1-specific activator SRT2104 combined with LDHA inhibitor oxalate has a significant anti-tumor effect on gastric cancer cells, with limited adverse effects on normal gastric cells [151]. This suggests that combination therapy may be a breakthrough in future treatments.

Overall, only an in-depth understanding of lactation modifications can facilitate clinical applications and the discovery of more effective tumor therapeutic options.

## Data Availability

No datasets were generated or analysed during the current study.

## Abbreviations

TME: Tumor microenvironment
WGS: Whole Genome Sequencing
NMNAT1: Nicotinamide mononucleotide adenylyltransferase
KATs: Lysine acetyltransferases
GNAT: GCN5-related N-acetyltransferase
MYST: MOZ, YBF2, SAS2, and TIP60
CBP: CREB-binding protein
E.Coli: Escherichia coli
HBO1: Histone acetyltransferase binding to ORC1
NBS1: Nijmegen breakage syndrome 1
MRN: MRE11-RAD50-NBS1
HR: Homologous recombination
ACSS2: Acetyl-CoA synthetase
ERK: Extracellular signal-regulated kinase
Lactyl-CoA: Lactate-coenzyme A
AARS1: Alanyl-tRNA synthetase 1
PDHA1: Pyruvate Dehydrogenase E1 Subunit Alpha 1
CPT2: Carnitine Palmitoyltransferase 2
HDACs: Histone deacetylases
SIRT3: Sirtuin 3
DRF2: Double PHD fingers 2
PPP: Pentose phosphate pathway
YTHDF2: YTH N6-methyladenosine RNA-binding protein F2
PER1: Period circadian regulator 1
TP53: Tumor protein p53
ccRCC: Clear cell renal cell carcinoma
VHL: Von Hippel-Lindau
PDGERβ: Platelet-derived growth factor receptorβ
ICCA: Intrahepatic cholangiocarcinoma
TCGA: The cancer genome atlas
RHOF: Rho GTPase Rif
PKM2: Pyruvate kinase 2
EMT: Epithelial-mesenchymal transition
HCC: Hepatocellular carcinoma
DCBLD1: Discoidin, CUB, and LCCL domain-containing type I
PI3K/AKT: Phosphatidylinositol 3-kinase/Akt pathway
HIF: Hypoxia-inducible transcription factor
DLAT: Drolipoamide S-acetyltransferase
NSCLC: Non-small-cell lung cancer
HK2: Hexokinase 2
G6PD: Glucose-6-phosphate dehydrogenase
ULK1: UNC-51-like kinase
Vps34: Vacuolar sorting protein 34
LDHA: Lactate dehydrogenase
TFEB: Transcription factor EB
TNFSF9: Tumor necrosis factor superfamily member 9
IL-11: Interleukin-11
JAK2: Janus kinase 2
STAT3: Signal transducer and activator of transcription 3
NSCLC: Non-small cell lung cancer
POM121: Pore membrane protein 121
PD-L1: Programmed cell death-ligand 1
LOX: Lysyl oxidase
CAFs: Cancer-associated fibroblasts
VM: Vasculogenic mimicry
PCa: Prostate cancer
VEGFA: Vascular endothelial growth factor A
Sema3A: Semaphorin 3 A
KIAA1199: Cell migration-inducing protein, CEMIP
SLC25A29: Solute Carrier Family 25 Member 29
GP73: Golgi protein 73
GRR37: G protein-coupled receptor 37
VCAM1: Vascular cell adhesion molecule-1
KCNK1: Potassium Two Pore Domain Channel Subfamily K Member 1
CSCs: Cancer stem cells
LCSCs: Liver cancer stem cells
RUBCNL: Rubicon Like Autophagy Enhancer
XRCC1: X-ray cross complementing protein 1
YY1: Yin Yang 1
YBX1: The Y-box binding protein 1
PCK2: Phosphoenolpyruvate carboxykinase
IR: Lonizing radiation
PCA: Principal component analysis
LRGs: Lactylation-modified related genes
2-DG: 2-Deoxy-D-glucose
RJA: Royal Jelly Acid
DML: Demethylzeylasteral
PD-1: Programmed death receptor-1
HKL: Honokiol
CircRNAs: Circular RNAs
CAP: Cold atmospheric plasma
PFK-1: Phosphofructokinase
TTK: TTK protein kinase
BUB1B: BUB1 mitotic checkpoint serine/threonine kinase B
